# Multimodal Glioma Image Segmentation Using Dual Encoder Structure and Channel Spatial Attention Block

**DOI:** 10.3389/fnins.2020.586197

**Published:** 2020-10-28

**Authors:** Run Su, Jinhuai Liu, Deyun Zhang, Chuandong Cheng, Mingquan Ye

**Affiliations:** ^1^Institute of Intelligent Machines, Hefei Institutes of Physical Science, Chinese Academy of Sciences, Hefei, China; ^2^Science Island Branch of Graduate School, University of Science and Technology of China, Hefei, China; ^3^School of Engineering, Anhui Agricultural University, Hefei, China; ^4^Department of Neurosurgery, The First Affiliated Hospital of University of Science and Technology of China, Hefei, China; ^5^Division of Life Sciences and Medicine, University of Science and Technology of China, Hefei, China; ^6^Anhui Province Key Laboratory of Brain Function and Brain Disease, Hefei, China; ^7^School of Medical Information, Wannan Medical College, Wuhu, China

**Keywords:** medical image fusion, glioma segmentation, fully convolutional neural networks, DES, CSAB, F-S-Net

## Abstract

Multimodal medical images provide significant amounts of complementary semantic information. Therefore, multimodal medical imaging has been widely used in the segmentation of gliomas through computational neural networks. However, inputting images from different sources directly to the network does not achieve the best segmentation effect. This paper describes a convolutional neural network called F-S-Net that **f**uses the information from multimodal medical images and uses the semantic information contained within these images for glioma **s**egmentation. The architecture of F-S-Net is formed by cascading two sub-networks. The first sub-network projects the multimodal medical images into the same semantic space, which ensures they have the same semantic metric. The second sub-network uses a dual encoder structure (DES) and a channel spatial attention block (CSAB) to extract more detailed information and focus on the lesion area. DES and CSAB are integrated into U-Net architectures. A multimodal glioma dataset collected by Yijishan Hospital of Wannan Medical College is used to train and evaluate the network. F-S-Net is found to achieve a dice coefficient of 0.9052 and Jaccard similarity of 0.8280, outperforming several previous segmentation methods.

## 1. Introduction

Gliomas, which arise from the canceration of gliocyte in the brain and myelon, are the most common form of cancer in the skull, accounting for 80% of malignant brain tumors (Ostrom et al., 2014). The incidence ranges from 3 to 8 per 100,000 people and the fatality rate is high. Hence, the early diagnosis and treatment of gliomas are very important. The presence of gliomas can also cause complications such as increased intracranial pressure, brain edema, brain hernia, and psychosis. The size, location, and type of a glioma are determined by segmenting the affected region from other normal brain tissue. Accurate segmentation plays an important role in the diagnosis and treatment of gliomas. However, manual delineation practices not only require significant anatomical knowledge, but are also expensive, time consuming, and inaccurate. The automatic segmentation of gliomas would allow doctors to detect the growth of brain tumors earlier and provide additional information for the generation of treatment plans. Bi et al. (2019) believed that artificial intelligence could improve the role of current standard diagnostic imaging technology by refining the preoperative classification of brain tumors above the level achievable by experts. Automatic segmentation based on computer-assisted intervention provides a steady solution for the treatment of gliomas, and is an effective tool in reducing the time required for the accurate detection, location, and delineation of tumor regions. Hence, it is necessary to automatically segment gliomas from medical images.

In recent years, methods based on deep learning (LeCun et al., 2015) have made significant breakthroughs in image classification (Krizhevsky et al., 2012; Rawat and Wang, 2017), image segmentation (Badrinarayanan et al., 2017; Garcia-Garcia et al., 2017), object detection (Ren et al., 2015; Zhao et al., 2019), object tracking (Li et al., 2018; Ristani and Tomasi, 2018), image captioning (Anderson et al., 2018; Hossain et al., 2019), and other fields (Hu et al., 2020). These breakthroughs have promoted the development of deep learning methods in the field of medical image analysis (Litjens et al., 2017; Altaf et al., 2019; Esteva et al., 2019). One of the best-known architectures for medical image segmentation is U-Net, initially proposed by Ronneberger et al. (2015), in which the backbone is a fully convolutional network (FCN) (Long et al., 2015). U-Net has received widespread attention from researchers in the field of medical image segmentation, and many improvements to U-Net have since been proposed (Alom et al., 2018; Oktay et al., 2018; Zhou et al., 2018). For example, Milletari et al. (2016) proposed V-Net for processing 3D medical images, whereby residual learning is employed to improve the convergence speed of the network and random nonlinear transformation and histogram matching are used for data augmentation. Milletari et al. also proposed the dice loss technique based on dice coefficients. Cheng et al. (2019) obtained a multilevel glioma segmentation network by combining an attention mechanism and atrous convolution with 3D U-Net. Chen et al. (2018b) used 3D U-Net and separable 3D convolution to build a separable 3D U-Net architecture. A multiscale masked 3D U-Net was proposed by Xu et al. (2018). The input to their network is a superimposed multiscale map, and multiscale information is obtained from the 3D ASPP layer.

Although methods based on deep learning have been widely used in this field, the current approaches have some disadvantages. Usually, researchers combine multimodal or multisequence medical images to obtain better segmentation accuracy (Kamnitsas et al., 2017b; Chen et al., 2018b; Xu et al., 2018; Zhao et al., 2018; Cheng et al., 2019). The multimodal medical images are input directly into the network for learning. However, the semantic conflicts between multimodal medical images cannot be completely avoided, and these may have a certain impact on the segmentation results. The method of image fusion can integrate valuable information from multimodal medical images, and the fusion results are typically more comprehensive than the original images (Liu et al., 2017). To date, there have been few reports on the segmentation of gliomas based on multimodal medical image fusion.

Another disadvantage of existing methods is that U-Net variants do not improve the basic architecture of U-Net. In particular, the features of the medical images are extracted by a single encoder. This means there may be a loss of feature information. Therefore, it is necessary for networks to obtain and retain more useful features.

In this paper, we propose F-S-Net, which combines image fusion technology to obtain images with richer semantic information. F-S-Net consists of two sub-networks: a **f**usion sub-network and a **s**egmentation sub-network. The fusion sub-network projects images obtained from computed tomography (CT) and magnetic resonance imaging (MRI) into the same semantic space for fusion. Compared with the original images, the fused image contains more semantic information for segmentation. To improve the segmentation performance, the segmentation sub-network uses a dual encoder structure (DES) and a channel spatial attention block (CSAB) to perform image segmentation. Based on the U-Net architecture, DES and CSAB use different sizes of convolution kernel to extract more effective features and focus on the lesion area. In the process of skip-connection, a 1×1 convolution and a concatenation operation are used to achieve better feature fusion. This method is conducive to feature extraction and utilization, and can achieve good performance. DES and CSAB are integrated into the networks based on the U-Net framework, and are found to improve the segmentation result. Experiments show that the cascaded networks proposed in this paper achieve better performance than existing approaches.

The contributions of this study are as follows:

1. A DES is constructed by increasing the width of the encoder. The proposed structure uses convolution kernels of different sizes to extract more effective features from images.
2. Our CSAB is constructed by combining channel attention and spatial attention mechanisms in the U-Net architecture. The proposed attention mechanism can be easily integrated into other networks that use the U-Net framework.
3. The proposed F-S-Net is formed by combining two sub-networks. One sub-network fuses CT and MRI images to enhance the semantic information of the images, while the other is used to segment gliomas accurately from the fused image.
4. Clinical glioma imaging data were collected from Yijishan Hospital of Wannan Medical College. The labels of each image were annotated by professional medical staff. The collected dataset provides a valuable tool for further research.
5. Extensive comparison experiments were conducted based on the collected dataset to demonstrate that the proposed method obtains the best segmentation performance among several deep segmentation methods.

## 2. Related Work

Convolutional neural networks (CNNs) are a common architecture for glioma segmentation, especially the encoder–decoder model.

Wang et al. (2017) trained each tumor sub-region by using networks with similar architectures and cascading these networks. The input to each network was the output from the previous network. However, some loss of global information might be caused by the way the gliomas are progressively segmented. Kamnitsas et al. (2017a) reported better results using ensembles of multiple models and architectures (EMMA). In particular, EMMA combined the DeepMedic (Kamnitsas et al., 2017b), FCN, and U-Net models and synthesized their segmentation results. The strong performance of EMMA helped Kamnitsa et al. to win the BraTS Challenge in 2017. However, EMMA does not offer end-to-end training, and the final result is affected by the accumulation of errors. Unlike most researchers, Isensee et al. (2018) demonstrated that competitive performance could be achieved with a few minor modifications to a generic U-Net. They reduced the number of feature maps before sampling from the decoder, and used additional training data to produce some improvements in terms of tumor enhancement. Myronenko (2018) won the BraTS 2018 challenge with a segmentation network based on the encoder–decoder architecture. An asymmetric encoder is used to extract features, and then two decoders segment the brain tumor and reconstruct the input image, respectively. The first decoder outputs the segmentation results from three tumor sub-regions, while the second uses a variational auto-encoder (VAE) to reconstruct the input image. The VAE branch only reconstructs the input images during the training stage. Jiang et al. (2019) achieved the best results in the 2019 BraTS challenge. They proposed a U-Net-based cascade network that is divided into two stages. In the first stage, a variant of U-Net produces an unshaped result. In the second stage, improved performance is obtained by increasing the width of the decoder. In fact, their network uses two decoders that are structurally similar, but have some differences in their up-sampling procedures: one decoder uses deconvolution while the other uses trilinear interpolation. Although multimodal medical imaging has been widely used in glioma segmentation, few researchers have considered the processing of multimodal medical images. This is a clear gap in the research, as the results might be affected by the different semantic information contained in multimodal medical images.

## 3. Methods

This section describes the proposed F-S-Net architecture in detail. F-S-Net consists of two sub-networks, a fusion sub-network and a segmentation sub-network. The fusion sub-network uses multimodal images to obtain more detailed medical images with a wealth of semantic information. After processing the corresponding CT and MRI images, the fusion results are input to the segmentation sub-network. The segmentation sub-network uses a dual encoder architecture to extract detailed features from the lesion area. Different sizes of convolutional kernel are used to process images on parallel paths. At the same time, an attention mechanism is integrated into the CSAB module among the skip-connection processing. The final result is obtained by segmenting the fused results.

### 3.1. F-S-Net

**Figure 1:**
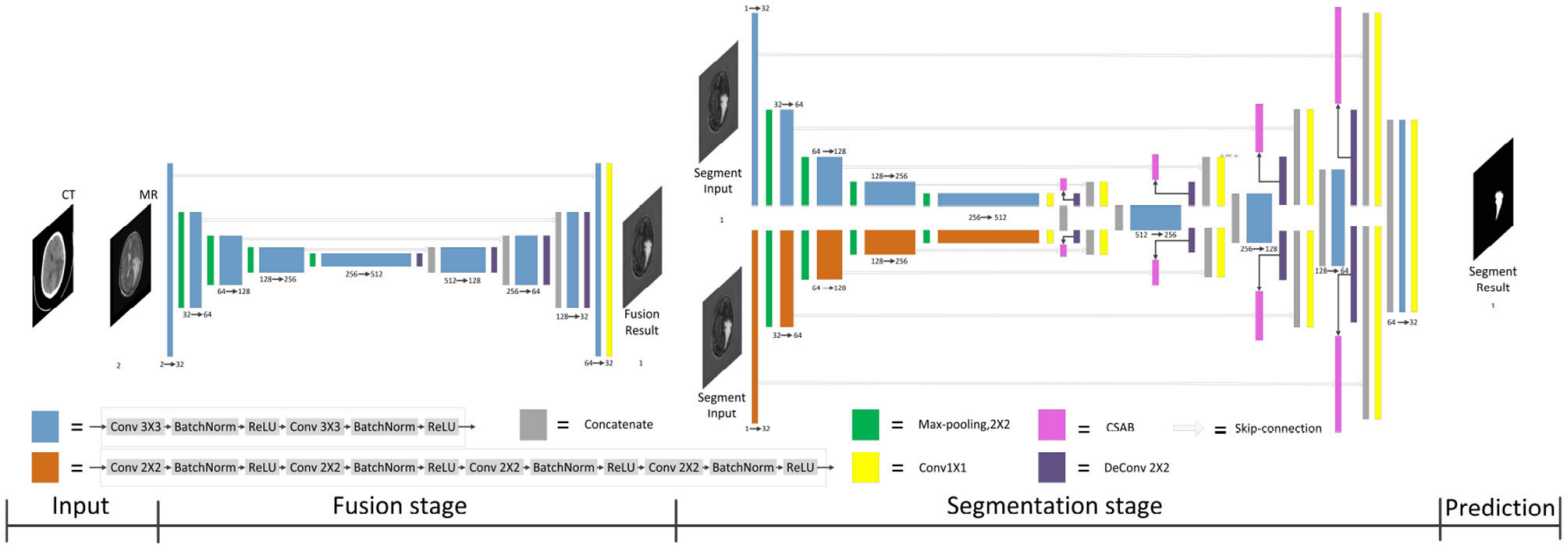
Architecture of the proposed F-S-Net for glioma segmentation. The corresponding CT and MRI images are processed by the fusion sub-network. The fusion images are copied into the segmentation sub-network, which uses convolution kernels of different sizes to process images on parallel paths. The final results of segmentation are output by the decoder.

Multimodal medical images have been widely used in medical image analysis tasks. As multimodal images contain different semantic information, image fusion technology is used to map the semantic information from the multimodal images to the same semantic space, including image structure information and edge information. Therefore, F-S-Net incorporates medical image fusion technology. The proposed network architecture is shown in Figure 1.

**Figure 2:**
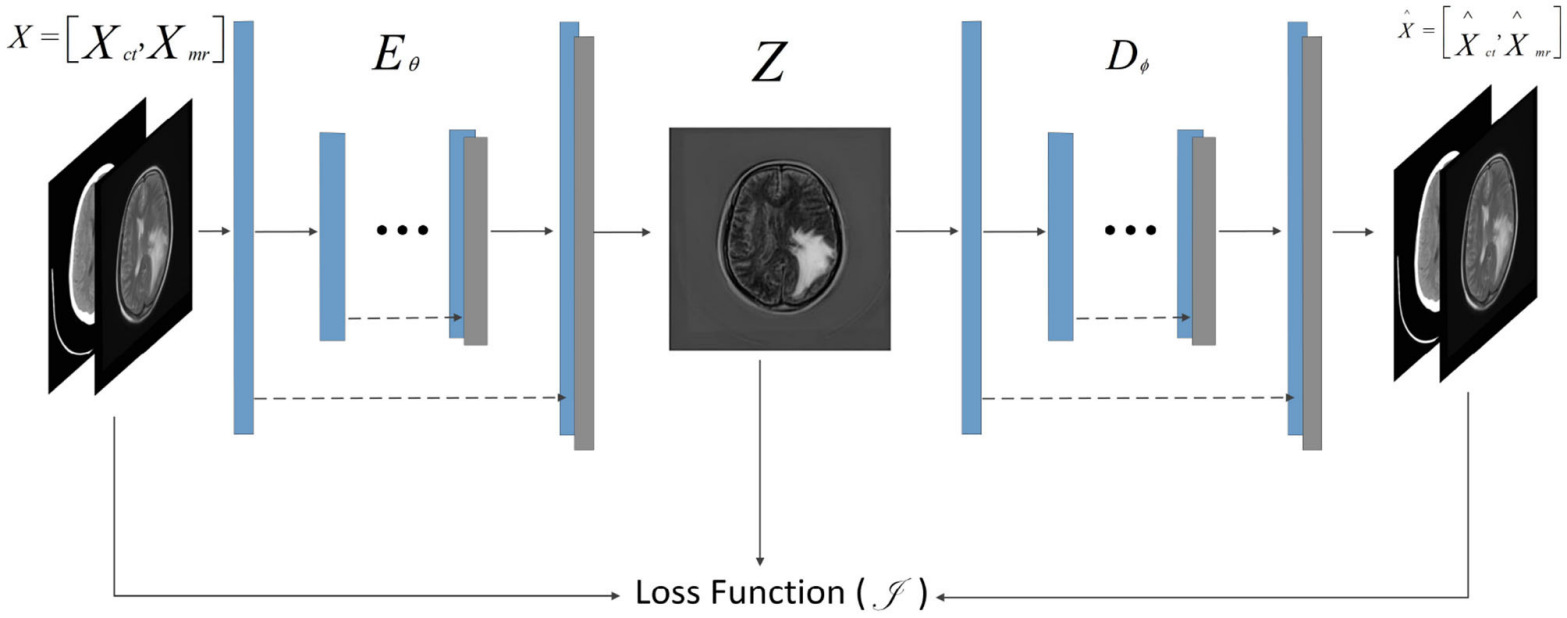
Architecture of fusion sub-network for glioma segmentation. The input size of the network is 256×256. The *E*_*θ*_ and *D*_*ϕ*_ are follows the structure of U-Net. The *D*_*ϕ*_ is used only during training to reconstruct the input.

**Figure 3:**
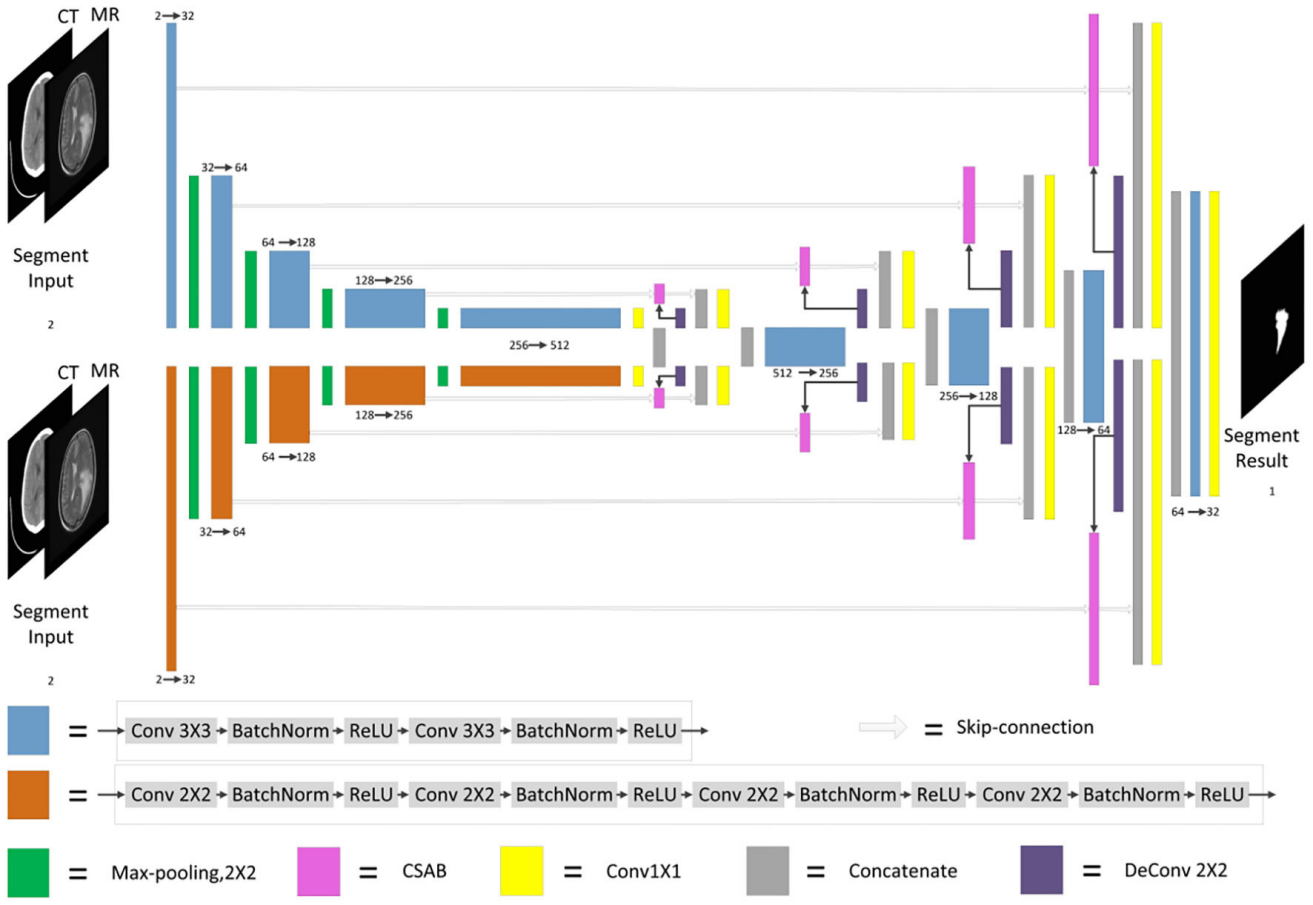
Architecture of segmentation sub-network for glioma segmentation. The input size of the network is 256×256. Each different box denotes the different operations. The number of channels is denoted on the boxes. The parallel pathways process each feature using different sizes of convolution kernel, which are combined at the end of the encoder.

F-S-Net is divided into two stages. In the first stage, the fusion sub-network is used to fuse CT and MRI images. As the semantic information from various multimodal images is combined, this process provides more detailed medical images for segmentation networks. In the second stage, the fused image is input into the segmentation sub-network. The CSAB and DES modules are used in the segmentation sub-network based on the U-Net architecture. Figure 2 shows the structure of the fusion sub-network (Fan et al., 2019). The *E*_*θ*_ and *D*_*ϕ*_ of fusion sub-network are follows the structure of U-Net. *E*_*θ*_ is used to generate the fusion results. *D*_*ϕ*_ is used to reconstruct the input. The loss value is determined by the input, fusion results, and reconstruction results. The loss function of the fusion sub-network has been modified by us. The details of the loss function are described in section 3.4. *D*_*ϕ*_ is used during the training stage. The segmentation sub-network architecture is a typical encoder–decoder structure, as shown in Figure 3. The segmentation sub-network consists of two encoders (left side) and a decoder (right side). The two encoders use convolution kernels of different sizes. In the skip-connection process, the attention mechanism is used to enable the network to extract the features of a specific area and perform feature fusion. The decoder is the same as in U-Net. The network takes input images of 256×256 pixels, and outputs images of the same size. The network can obtain more comprehensive and consistent medical images, and perform better segmentation tasks, after multimodal image fusion. The results are generated by minimizing the loss value.

### 3.2. Channel Spatial Attention Block

**Figure 4:**
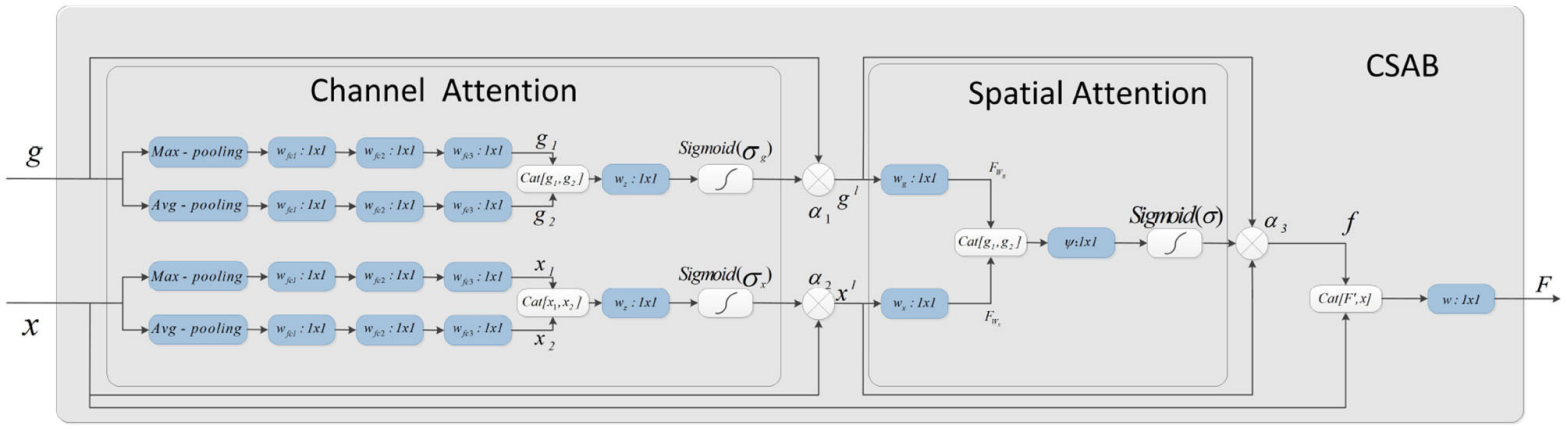
Schematic of the proposed CSAB. CSAB is composed of Channel Attention Block (CAB) and Spatial Attention Block (SAB). CAB applies channel attention weight to *x* from encoder part and *g* from upsampled, respectively. *x*^*l*^ and *g*^*l*^ are the feature maps of CAB output. *x*^*l*^ and *g*^*l*^ are fused in the spatial attention block. Spatial attention weights are applied to the result of feature fusion. *f* is the feature maps of SAB output. *F* is the feature map by CSAB output.

The attention mechanism is derived from the study of human vision. In computer vision, the attention mechanism allows the system to ignore irrelevant information and focus on important information. Combining channel attention, spatial attention, and the structural features of U-Net gives the CSAB module. This module enhances the salient features of the up-sampling process by applying an attention weight to the high- and low-dimensional features. The proposed structure is shown in Figure 4. The input feature maps *x* and *g* are scaled using the attention coefficient (α_3_) computed in CSAB. Areas of concern are selected by analyzing the different types of attention weights provided by *x* and *g*.

Given an intermediate feature map *x, g* ∈ *R*^*C*×*H*×*W*^ as input, CSAB obtains two intermediate 1D channel attention weights α_1_, α_2_ ∈ *R*^*C*×1×1^ and an intermediate 2D spatial attention weight α_3_ ∈ *R*^1×*H*×*W*^. Figure 4 describes the calculation for each attention module. The overall attention process can be summarized as:

(1) $$\begin{array}{l} g^{l}=\alpha_{1}\left(g\right)⊗g \end{array}$$

(2) $$\begin{array}{l} x^{l}=\alpha_{2}\left(x\right)⊗x \end{array}$$

(3) $$\begin{array}{l} f=\alpha_{3}\left(g^{l},x^{l}\right)⊗x^{l}⊗g^{l} \end{array}$$

(4) $$\begin{array}{l} F=w\left(Cat\left[f,x\right]\right)+b \end{array}$$

where ⊗ denotes element-wise multiplication. *F* is the final output obtained by 1×1 convolution after fusing *f* and feature *x*.

#### 3.2.1. Channel Attention Block

The channel attention weight is produced from high- and low-dimensional features using the relationship among the features. Four different spatial context descriptions, *g*_*max*_, *g*_*avg*_, *x*_*max*_, and *x*_*avg*_, are obtained using average pooling and maximum pooling operations on the feature map. These four characteristics are entered into a small network for further processing. The output feature vectors of the small network are merged using a concatenation operation. Finally, the channel attention weights α_1_*(g)* and α_2_*(x)* are obtained after the dimension has been reduced by 1×1 convolution. The channel attention is calculated as follows:

(5) $$\begin{array}{l} g_{max}=MaxPool\left(g\right) \end{array}$$

(6) $$\begin{array}{l} g_{avg}=AvgPool\left(g\right) \end{array}$$

(7) $$\begin{array}{l} x_{max}=MaxPool\left(x\right) \end{array}$$

(8) $$\begin{array}{l} x_{avg}=AvgPool\left(x\right) \end{array}$$

(9) $$\begin{array}{l} g_{1}=w_{fc3}\left(w_{fc2}\left(w_{fc1}\left(g_{max}\right)+b_{fc1}\right)+b_{fc2}\right)+b_{fc3} \end{array}$$

(10) $$\begin{array}{l} g_{2}=w_{fc3}\left(w_{fc2}\left(w_{fc1}\left(g_{avg}\right)+b_{fc1}\right)+b_{fc2}\right)+b_{fc3} \end{array}$$

(11) $$\begin{array}{l} x_{1}=w_{fc3}\left(w_{fc2}\left(w_{fc1}\left(x_{max}\right)+b_{fc1}\right)+b_{fc2}\right)+b_{fc3} \end{array}$$

(12) $$\begin{array}{l} x_{2}=w_{fc3}\left(w_{fc2}\left(w_{fc1}\left(x_{avg}\right)+b_{fc1}\right)+b_{fc2}\right)+b_{fc3} \end{array}$$

(13) $$\begin{array}{l} \alpha_{1}\left(g\right)=\sigma_{g}\left(w_{z}\left(Cat\left[g_{1},g_{2}\right]\right)+b_{z}\right) \end{array}$$

(14) $$\begin{array}{l} \alpha_{2}\left(x\right)=\sigma_{x}\left(w_{z}\left(Cat\left[x_{1},x_{2}\right]\right)+b_{z}\right) \end{array}$$

where σ_*g*_ and σ_*x*_ denote the sigmoid function, $W_{fc1}\in R^{C/8\times 1\times 1}$, $W_{fc2}\in R^{C/8\times 1\times 1}$, $W_{fc3}\in R^{C\times 1\times 1}$, and *W*_*z*_ ∈ *R*^*C*×1×1^. *W*_*fc*1_, *W*_*fc*2_, *W*_*fc*3_, and *W*_*z*_ denote the weight of each convolution. The rectified linear units (ReLU) activation function is followed by *W*_*fc*1_, *W*_*fc*2_, and *W*_*fc*3_.

#### 3.2.2. Spatial Attention Block

The spatial attention map is generated from α_1_*(g)* and α_2_*(x)* using the relationship among the features. The attention coefficient, α_3_ ∈ [0, 1], suppresses the expression of irrelevant regions in the input. In addition, the attention coefficient can highlight features that are useful for the task.

In the spatial attention block, the high- and low-dimensional features are subjected to 1×1 convolution to obtain two features: $F_{W_{g}}\in R^{C\times H\times W}$ and $F_{W_{x}}\in R^{C\times H\times W}$. The concatenation operation then performs feature fusion. Finally, the spatial attention map of $\alpha_{3}\in R^{1\times H\times W}$ is generated by 1×1 convolution. The output of the spatial attention block (SAB) is the element-wise multiplication of the input feature graph and the attention coefficient. The spatial attention is calculated as follows:

(15) $$\begin{array}{l} F_{W_{g}}=w_{g}\left(g^{l}\right)+b_{g} \end{array}$$

(16) $$\begin{array}{l} F_{W_{x}}=w_{x}\left(x^{l}\right)+b_{x} \end{array}$$

(17) $$\begin{array}{l} f=\alpha_{3}\left(g^{l},x^{l}\right)⊗x^{l}⊗g^{l}=\sigma \left(\psi \left(Cat\left[F_{W_{g}},F_{W_{x}}\right]\right)+b\right)⊗x^{l}⊗g^{l} \end{array}$$

where σ denotes the sigmoid function. *W*_*g*_, *W*_*x*_, and ψ represent the convolution kernel weights, and *b*_*g*_, *b*_*x*_, and *b* are the bias terms.

### 3.3. Dual Encoder Structure

The DES is developed by extending the encoder of U-Net. Two different encoders are used to extract features from images, and the convolution kernel size of the two encoders is different. One encoder has a convolution kernel size of 3×3, while the other has a convolution kernel size of 2×2. The encoder with a convolution kernel size of 3×3 is consistent with U-Net. Each layer consists of two 3×3 convolutions, followed by batch normalization (BN) and ReLU activation. The encoder with a convolution kernel of 2×2 is different from that of U-Net. Each layer consists of four 2×2 convolutions, each followed by BN and ReLU activation. The padding of the four 2×2 convolutions is 0101. The number of initial filters is 32. More feature information is obtained from images that use convolution kernels of different sizes. In addition, more significant information will be input to the decoder through the parallel paths design.

**Figure 5:**
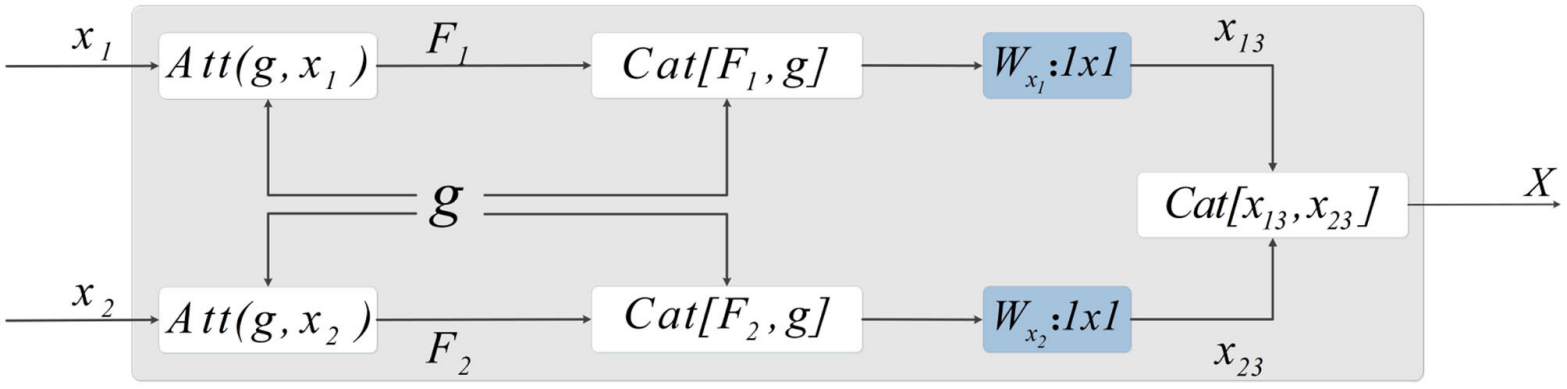
Method of skip-connection. First, the attention weight is applied by CSAB to the output characteristics of the encoder. The features obtained from the up-sampling are then fused with the features after applying the attention weight. Subsequently, 1×1 convolution is used to reduce the dimension of the fused features. The final output is obtained by concatenating the two features.

As the encoder has been expanded, it is necessary to fuse the features of each path when the features are input into the decoder. The output of CSAB is fused with the features obtained by up-sampling. Then, 1×1 convolution is used to reduce the dimension of the fused features. Finally, the processed features are input into the decoder. The two features from the encoder are processed separately. This approach is conducive to the integration of low- and high-dimensional information. The experimental results of the optimization procedure demonstrate the effectiveness of our structure. The structure designed in this study is shown in Figure 5.

Let *X*_1_ and *X*_2_ be features extracted by the encoder. *F*_1_ and *F*_2_ are the features output by CSAB, respectively, and *g* is the feature obtained after up-sampling. *F*_1_ and *F*_2_ are fused with *g*, and the features connected by skip-connection are subjected to 1×1 convolution for dimension reduction, resulting in *x*_13_ and *x*_23_. These two features are fused after dimensionality reduction to obtain *X*, which is input to the decoder. *X* is computed as follows:

(18) $$\begin{array}{l} X=Cat\left[x_{13},x_{23}\right] \end{array}$$

where *F*_1_, *F*_2_, *x*_13_, and *x*_23_ are given by:

(19) $$\begin{array}{l} F_{1}=Att\left(g,x_{1}\right) \end{array}$$

(20) $$\begin{array}{l} F_{2}=Att\left(g,x_{2}\right) \end{array}$$

(21) $$\begin{array}{l} x_{13}=W_{x_{1}}\left(Cat\left[F_{1},g\right]\right)+b_{1} \end{array}$$

(22) $$\begin{array}{l} x_{23}=W_{x_{2}}\left(Cat\left[F_{2},g\right]\right)+b_{2} \end{array}$$

DES has two advantages. First, the convolution kernels of the two encoders are 3×3 and 2×2, respectively. This strategy can extract more different features, which is beneficial to the segmentation task. Secondly, the features processed during the skip-connection ensure more complete information fusion. We have not made any major changes to the U-Net architecture. Therefore, our DES can be extended to most networks that are based on the U-Net architecture.

### 3.4. Loss Function

The loss function consists of three terms:

(23) $$\begin{array}{l} L_{total}=0.02*\left(L_{MSE}+L_{SSIM}\right)+L_{BCE} \end{array}$$

*L*_*MSE*_ is the mean squared error (MSE) loss between the reconstructed output *I*_*i*_ and the input image *O*_*i*_:

(24) $$\begin{array}{l} L_{MSE}=\frac{1}{N}\overset{N}{\underset{i=1}{\sum }}{\left(I_{i}-O_{i}\right)}^{2} \end{array}$$

where *N* is the number of epochs.

*L*_*SSIM*_ is calculated as:

(25) $$\begin{array}{l} L_{SSIM}=\frac{1}{N}\overset{N}{\underset{i=1}{\sum }}\left(1-SSIM\left(O_{i},F_{i}\right)\right) \end{array}$$

where SSIM(·) represents the structural similarity between two images (Wang et al., 2004). *F*_*i*_ represents the fused image.

*L*_*BCE*_ is the binary cross-entropy (BCE) loss applied to the segmentation output *P*_*i*_ and the segmentation mask *T*_*i*_:

(26) $$\begin{array}{l} L_{BCE}=-\frac{1}{N}\overset{N}{\underset{i=1}{\sum }}\left(T_{i}\text{ log}\left(P_{i}\right)+\left(1-T_{i}\right)\text{log}\left(1-P_{i}\right)\right) \end{array}$$

**Figure 6:**
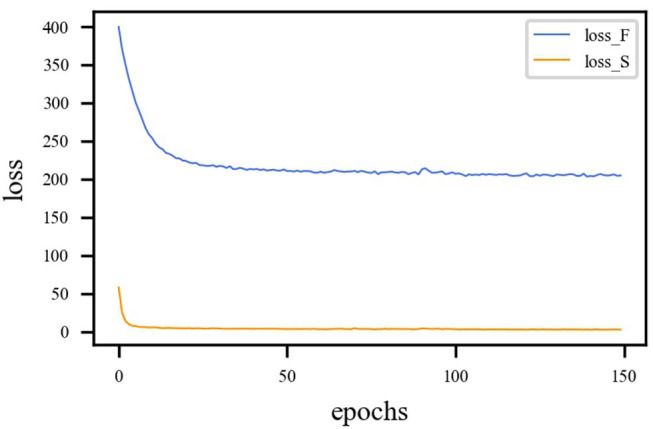
Loss curve of different sub-network. The blue line represents the loss function curve of the fusion sub-network. The orange line represents the loss function curve of the segmentation sub-network. Fusion loss is bigger than segmentation loss. Therefore, weights must be applied to balance the two sub-networks.

*L*_*MSE*_ and *L*_*SSIM*_ are the loss functions of the fusion sub-network, and *L*_*BCE*_ is the loss function for the segmentation sub-network. Since calculations of loss function is different, the loss functions must be balanced. The proposed model is trained with η = 1 and γ = 1. η represents the loss weight of the fusion sub-network. γ represents the loss weight of the segmentation sub-network. The loss curves are shown in Figure 6, from which we can learn that fusion loss is bigger than segmentation. To balance the loss weights between fusion and segmentation sub-networks, the loss weight in Equation (23) are set to η = 0.02 and γ = 1.

## 4. Results

### 4.1. Experimental Environment

A 12 GB NVIDIA Titan X (Pascal) was used for training and evaluation. The system was running Windows 10 with an Intel Xeon CPU with 64 GB RAM. The program was written on Pycharm and is based on the Pytorch (Paszke et al., 2019) framework.

### 4.2. Dataset

**Figure 7:**
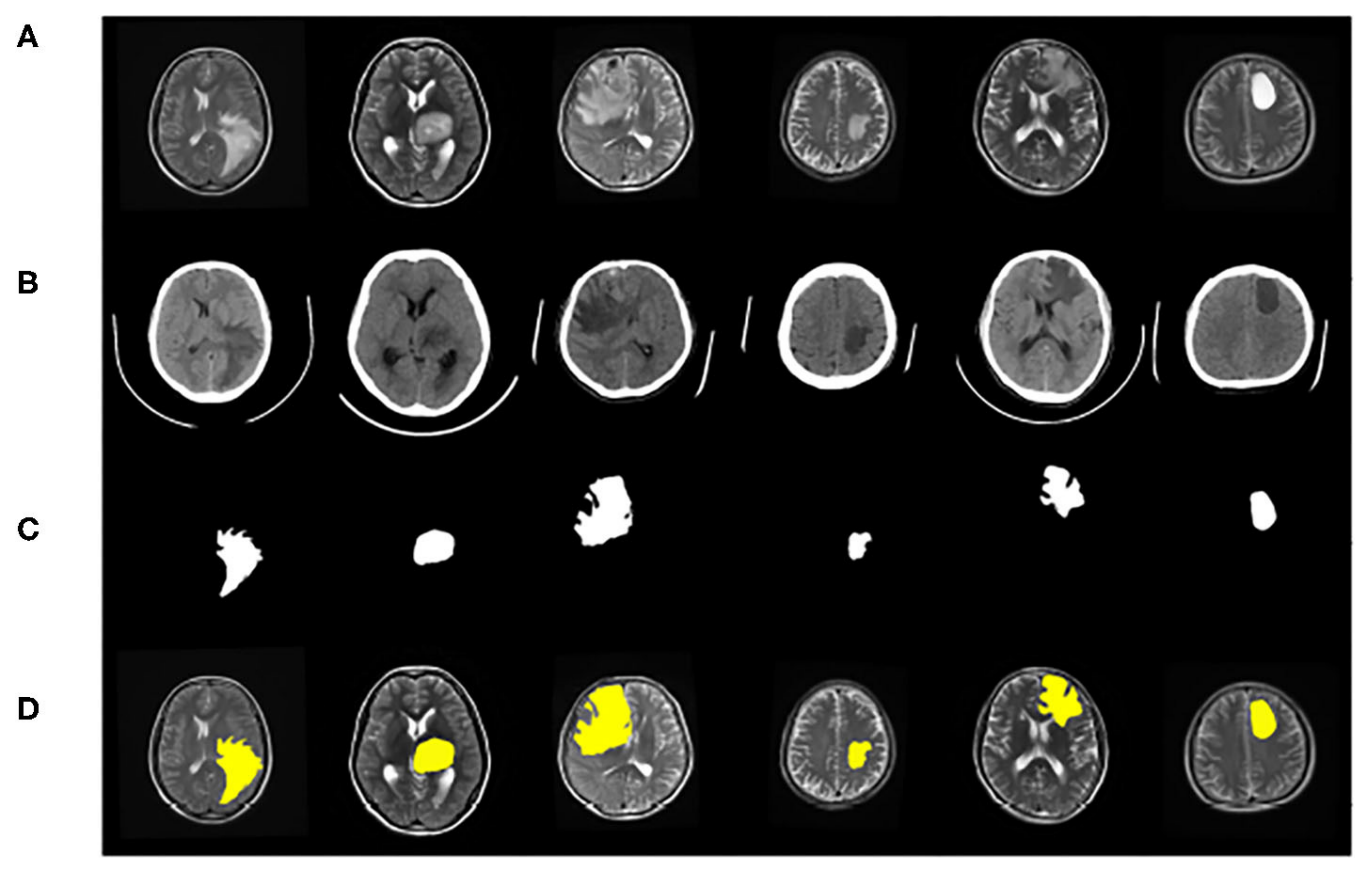
Example of image modalities and ground truth in the multimodal glioma dataset. **(A)** Shows a head scan CT. **(B)** Shows a T2-weighted MRI. **(C)** Shows the ground truth. **(D)** Shows the mergence result of **(B,C)**.

The dataset contains clinical imaging data from 26 patients with brain gliomas examined at Yijishan Hospital of Wannan Medical College. The clinical image data consist of CT and T2-weighted MRI scans from glioma patients, of which nine images were acquired from low-grade glioma patients and 17 images were obtained from high-grade glioma patients. These are brain scans before treatment. After slicing the data, 860 pieces of CT and MRI images were obtained. Registration was completed after slicing. In addition, an expert was invited from the First Affiliated Hospital of the University of Science and Technology of China to manual delineate the whole tumor area. The data are shown in Figure 7.

Data augmentation was used to improve the generalization ability and robustness of the models. As the image size may change after data augmentation, the images were resampled to 256×256 pixels. Finally, the dataset was randomly divided into a training dataset (60%), validation dataset (20%), and test dataset (20%).

### 4.3. Evaluation Measures

The accuracy rate (ACC), positive predictive value (PPV), Jaccard similarity (JS), and dice coefficient (DC) were used as evaluation indexes. These metrics were calculated as follows:

(27) $$\begin{array}{l} ACC=\frac{TP+TN}{TP+FP+TN+FN} \end{array}$$

(28) $$\begin{array}{l} PPV=\frac{TP}{TP+FP} \end{array}$$

(29) $$\begin{array}{l} JS=\frac{TP}{FP+TP+FN} \end{array}$$

(30) $$\begin{array}{l} DC=\frac{2*TP}{2*TP+FP+FN} \end{array}$$

where TP (true positive) represents the number of foreground pixels that are correctly classified as foreground (tumor region), TN (true negative) represents the number of background pixels that are correctly classified as background (non-tumor region), FP (false positive) represents the number of background pixels that are correctly identified as foreground, and FN (false negative) represents the number of foreground pixels that are incorrectly classified as background.

ACC is used to represent the classification accuracy of the classifier. PPV represents the proportion of true positives in all positive cases. JS reflects the ratio of the common area of the matched element to the split result. Any imprecise segmentation, whether under- or over-segmentation, will cause the JS to decrease. DC calculates the similarity between the prediction results and the ground truth to evaluate the performance of the model.

### 4.4. Training Optimization

**Figure 8:**
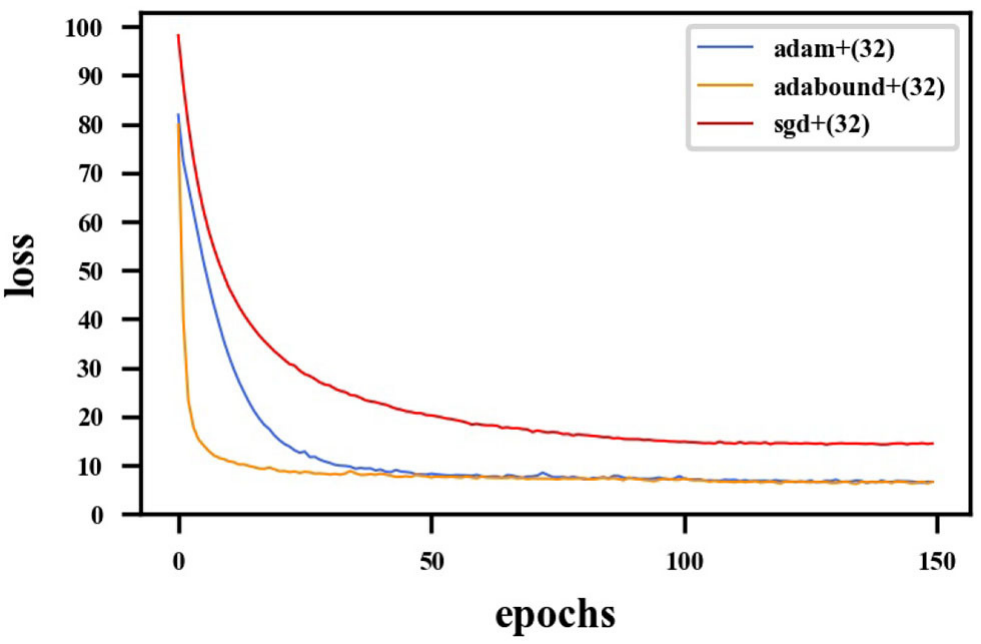
Loss curve for F-S-Net with different optimizers. The red line represents loss function curve of the SGD optimizer. The blue line represents the loss function curve of the Adam optimizer. The orange line represents the loss function curve of the Adabound optimizer. The optimizer of Adabound has the fastest rate of convergence.

First, the appropriate numbers of optimizers and convolution kernels were determined. Stochastic gradient descent (SGD) (Robbins and Monro, 1951) has been widely applied in the field of deep learning, while adaptive moment estimation (Kingma and Ba, 2014) offers better optimization performance. Adabound (Luo et al., 2019) dynamically crops the learning rate so that the algorithm is closer to Adam in the early stages of training and closer to SGD at the end. For CNNs, the receptive field and number of channels on the receptive field determine the performance of the network. The convolution kernels considered in the experiments had the following structures: (16) 1-16-32-64-128-256-128-64-32-16-1; (32) 1-32-64-128-256-512-256-128-64-32-1. Four experimental groups were examined in the experiments: (1) Adam + (32), (2) Adabound + (32), (3) SGD + (32), and (4) Adabound + (16). The number of training epochs was set to 150, the batch size was set to 4, the weight decay was set to 5×10^−8^, and the learning rate decreased by 0.1 after the 100th epoch. The experimental results are presented in Table 1. The loss curve is shown in Figure 8. In Figure 8, Adabound converges faster than the other optimizers. On the independent test dataset, the DCs of SGD, Adabound, and Adam are 0.8839, 0.8975, and 0.8922, respectively. Based on these results, Adabound and structure (32) were used in subsequent experiments.

**Table 1:** JS and DC for F-S-Net with different numbers of kernels and optimizers.

**Number of convolution kernels**	**JS**	**DC**
Adam + (32)	0.8070	0.8922
Adabound + (32)	0.8172	0.8975
SGD + (32)	0.7936	0.8839
Adabound + (16)	0.8040	0.8902

Convolution kernels of different sizes have different receptive fields. The convolution kernel size of one encoder was kept the same as that in U-Net, while the convolution kernel size of the other encoder was modified as follows: (1) The 3×3 convolution of the amplified path was replaced by 5×5 convolution. (2) The two 3×3 convolutions were kept unchanged. (3) The 3×3 convolution of the amplified path was replaced by two 2×2 convolutions. Note that the padding is different when using 2×2 convolution. The experimental results presented in Table 2 show that replacing a set of 3×3 convolutions with a set of 2×2 convolutions produces a better effect.

**Table 2:** DC and JS for F-S-Net with different sizes of kernels and optimizers in the encoder–decoder for test dataset.

**Sizes of convolution kernel**	**JS**	**DC**
3×3-3×3	0.8172	0.8975
3×3-5×5	0.8226	0.9019
3×3-2×2(0101)	0.8234	0.9023
3×3-2×2(1010)	0.8226	0.9014

*0101 and 1010 are the settings for the padding in each 2×2 convolution block*.

**Figure 9:**
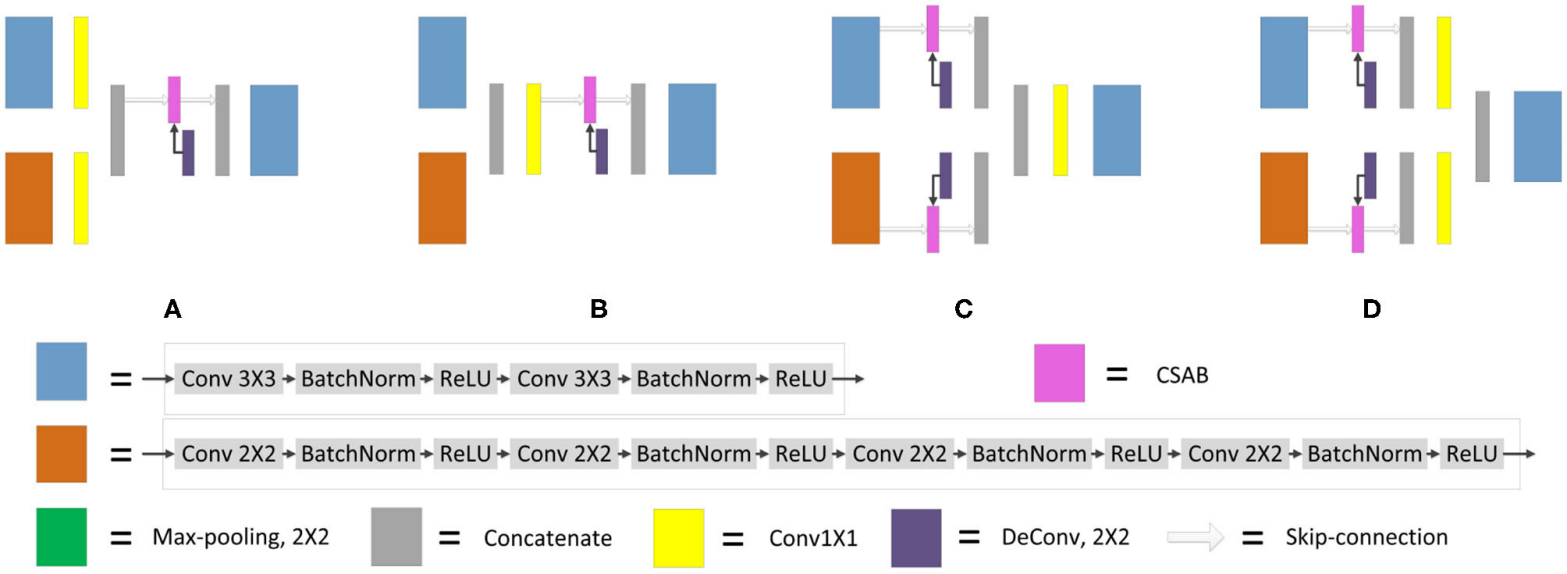
Different types of skip-connection. The skip-connection of F-S-Net is divided into three parts: (**Cu**) CSAB and up-sampling. (**1×1**) 1×1 convolution. (**Cat**) concatenation. **(A)** (**1×1**) + (**Cat**) + (**Cu**). **(B)** (**Cat**) + (**1×1**) + (**Cu**). **(C)** (**Cu**) + (**Cat**) + (**1×1**). **(D)** (**Cu**) + (**1×1**) + (**Cat**).

It is necessary to modify the skip-connection to adapt to the inputs of the two encoders. An increase in skip-connection input would inevitably require feature fusion and dimensionality reduction. The order of 1×1 convolution and feature fusion may affect the performance of the network. Therefore, the four different structures shown in Figure 9 were constructed.

Experiments were performed using the above four structures. The final experimental results are presented in Table 3, showing that better results are obtained by the skip-connection and dimension reduction of the two paths, respectively.

**Table 3:** DC and JS for F-S-Net with different types of skip-connection.

**Skip-connection type**	**JS**	**DC**
a	0.8234	0.9023
b	0.8019	0.8883
c	0.8109	0.8947
d	0.8280	0.9052

### 4.5. Ablation Analysis of Proposed Methods

**Figure 10:**
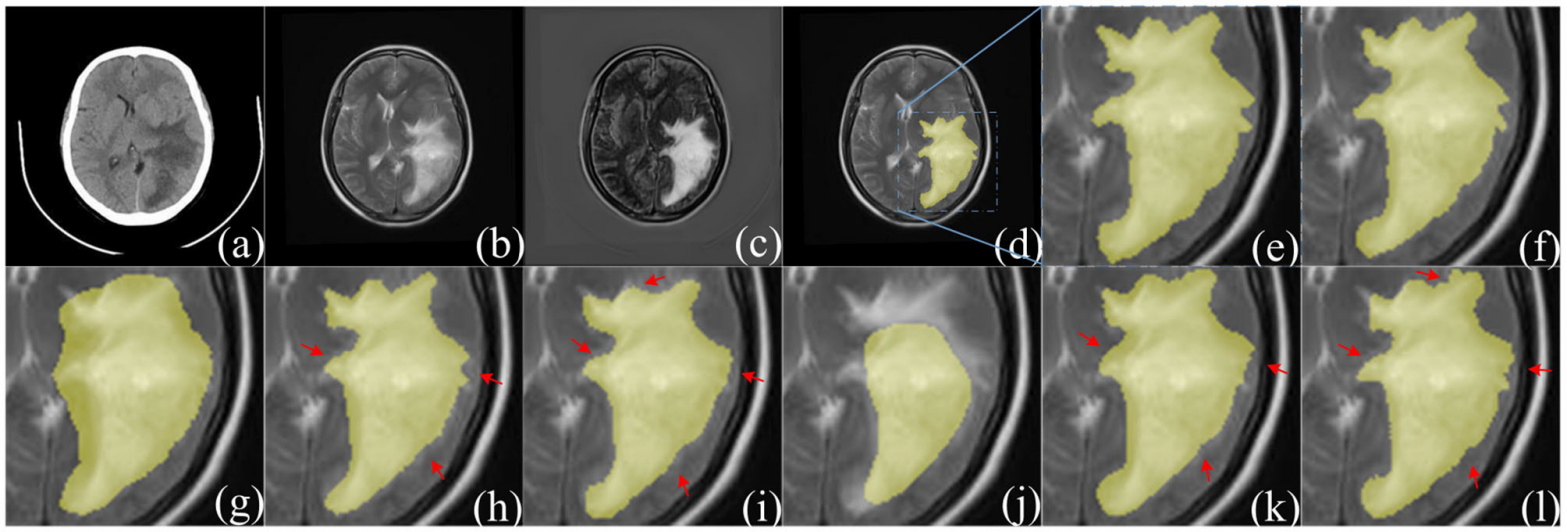
Comparison of segmentation results between F-S-Net and other networks. **(a,b)** source images before fusion, **(c)** fusion result. Compared with **(a,b)**, the features in **(c)** are more obvious. The ground-truth glioma segmentation **(e)** is highlighted in **(d)**. Similarly, other model predictions are compared with those of F-S-Net **(f)**. **(g–l)** Are the results given by FCN8s, SegNet, DeeplabV3+, U-Net, R2U-Net, and AttU-Net, respectively. The missed dense predictions by other models are highlighted with red arrows.

The experimental results of the proposed structures with non-fusion and fusion were compared. It is clear that the improved structure and combination of modules are effective in enhancing the glioma segmentation results. The hyperparameters were set according to the previous optimization experiment. The training and testing samples for the experiment were taken from the glioma dataset. The fusion results in Figure 10 clearly represent the overall area of the tumor, which makes the image features more obvious. The glioma can be accurately segmented and the network captures the specific outline and edge details of the lesion area in the image. Table 4 presents the experimental results from using the proposed architecture.

**Table 4:** Evaluation metric for ablation analysis of our methods with test dataset.

**Model**	**ACC**	**PPV**	**JS**	**DC**
U-Net (Ronneberger et al., 2015)	0.9916	0.8001	0.7656	0.8656
DECSAU-Net (Ours)	0.9938	0.8624	0.8193	0.8994
F-S-Net (Ours)	0.9943	0.9054	0.8280	0.9052

DECSAU-Net is the segmentation sub-network in F-S-Net. When the proposed modules are removed, the network architecture is the same as the standard U-Net. Comparing the network models with and without DES and CSAB, it can be seen that the inclusion of DES and CSAB results in better performance. The PPV of DECSAU-Net is about 0.0623 higher than that of U-Net. The JS and DC values are about 0.0537 and 0.0338 higher, respectively. A comparison with U-Net shows that DES and CSAB improve the results of U-Net.

The results achieved with non-fusion and fusion approaches are now compared. The DC of the fused image is about 0.0058 higher than that of the image before fusion. The PPV of glioma segmentation after fusion is also higher at 0.9054. The difference in JS values shows that the result obtained after fusion is more similar to the ground truth. In general, the higher DC and JS values demonstrate that the segmentation is more accurate after fusion.

### 4.6. Comparison With Other Methods

Table 5 compares the performance of different network architectures with that of the proposed F-S-Net after normalizing and enhancing the glioma data on the same test dataset. Figure 10 shows the glioma segmentation results, which can be used to compare F-S-Net with other networks.

**Table 5:** Evaluation metrics for different network architectures.

**Model**	**ACC**	**PPV**	**JS**	**DC**
FCN8s (Long et al., 2015)	0.9885	0.7714	0.6980	0.8197
SegNet (Badrinarayanan et al., 2017)	0.9931	0.8428	0.8039	0.8890
DeeplabV3+ (Chen et al., 2018a)	0.9931	0.8328	0.8066	0.8914
U-Net (Ronneberger et al., 2015)	0.9916	0.8001	0.7656	0.8656
R2U-Net (Alom et al., 2018)	0.9932	0.8472	0.8040	0.8905
AttU-Net (Oktay et al., 2018)	0.9934	0.8586	0.8087	0.8932
DECSAU-Net (Ours)	0.9938	0.8624	0.8193	0.8994
F-S-Net (Ours)	0.9943	0.9054	0.8280	0.9052

Several medical image segmentation architectures (Ronneberger et al., 2015; Badrinarayanan et al., 2017; Alom et al., 2018; Chen et al., 2018a; Oktay et al., 2018) are outperformed by F-S-Net in both evaluations. The results in Table 5 indicate that F-S-Net is more effective for performing accurate glioma segmentation. Compared with other network architectures, our method is more conducive to the segmentation of lesions as it maps multimodal medical images into the same semantic space. The advantage of F-S-Net is that the fusion of multimodal images makes the semantic information more conspicuous, and DES and CSAB allow the network to achieve a better segmentation effect.

## 5. Discussion

Segmenting gliomas directly from CT or MRI images is a challenging task. In addition, the blurred edges of adjacent bones, blood vessels, or surgical packaging materials greatly increase the difficulty of segmentation.

Currently, most researchers directly input multimodal images into a network for learning. To the best of our knowledge, there are few reports on the segmentation of gliomas based on multimodal medical image fusion. To bridge this gap, F-S-Net has been proposed based on medical image fusion technology. Fusion and segmentation sub-networks are cascaded for end-to-end training, and two new structures, DES and CSAB, are proposed based on the structural characteristics of U-Net. The basic idea of F-S-Net is to use fusion technology to produce images with more semantic information for the segmentation network, so as to obtain better segmentation results. DES and CSAB extract more detailed features and force the network to focus on the lesion area. Our work builds on existing techniques, such as CT and MRI image fusion. Medical image fusion techniques are not specifically designed for the segmentation task, but can provide images with richer semantic information for segmentation.

The most important innovation described in this paper is the ability to perform the task of glioma segmentation using image fusion. In the field of medical image analysis, better performance is often achieved by combining different technologies. The results in Table 4 demonstrate the effectiveness of DES and CSAB, while those in Tables 4, 5 demonstrate the improvement offered by using fusion technology for segmentation. Our network has the following advantages. First, image fusion can enrich the information available by integrating information between multimodal medical images. This method improves the quality of the image and facilitates the segmentation task. Second, the convolution kernels of different sizes in DES allow the network to obtain richer features. This helps to focus attention on the area of interest, and then obtains a better segmentation effect. Third, CSAB makes the network focus on the lesion area by applying different attention weights to the features. Our method not only integrates the complementary information from different modalities, but also extracts more detailed features. The experimental results show that F-S-Net outperforms several existing methods.

In summary, our proposed method will be helpful in allowing clinicians to diagnose and treat gliomas. More detailed segmentation results provide doctors with more complete boundary information of the tumor, and can better guide the resulting operations. In addition, better segmentation contributes to the reconstruction of the image data, which can provide more information for future monitoring and treatment planning. Our method overcomes the problem of incomplete semantic information and achieves good performance. The combination of segmentation and other medical imaging technologies will be explored in the future. This may improve clinical guidance in the diagnosis and treatment of glioma patients.

## 6. Conclusion

Glioma segmentation is a challenging and significant task in medical image segmentation. Based on medical image fusion technology, a cascade network was proposed to automatically segment gliomas from CT and MRI images. Our network obtained a DC of 0.9052 on the test dataset. Experimental results show that the combination of image fusion and image segmentation is effective. Our model provides a new method and a new idea for glioma segmentation based on deep learning, and is beneficial to the clinical diagnosis and treatment of patients. The proposed network is not only applicable to the segmentation of gliomas, but could also be easily applied to other medical image segmentation tasks.

## Data Availability Statement

The datasets presented in this article are not readily available because, datasets are open in the future. Requests to access the datasets should be directed to Deyun Zhang, zhangdeyun2016@163.com.

## Author Contributions

RS and JL: conceptualization. RS, CC, and MY: data curation. RS: methodology, project administration, visualization, and writing (original draft). RS, JL, and DZ: validation and writing (review and editing). All authors have read and agreed to the published version of the manuscript.

## Conflict of Interest

The authors declare that the research was conducted in the absence of any commercial or financial relationships that could be construed as a potential conflict of interest.
